# Supplementary material for: Modeling glioblastoma heterogeneity as a dynamic network of cell states
Source: Mol Syst Biol. 2021 Sep 16;17(9):e10105. doi: 10.15252/msb.202010105 (PMC8444284; doi:10.15252/msb.202010105)
Supplement: Supplementary file 5 — Source Data for Figure 3 [file MSB-17-e10105-s001.zip › Figure3A_sourcedata/GSEA_3065/hallmarks_state4.GseaPreranked.1623416296068/gsea_report_for_na_pos_1623416296068.html]

Report for na\_pos 1623416296068 [GSEA]

| GS  follow link to MSigDB | GS DETAILS | SIZE | ES | NES | NOM p-val | FDR q-val | FWER p-val | RANK AT MAX | LEADING EDGE || 1 | HALLMARK\_OXIDATIVE\_PHOSPHORYLATION | Details ... | 152 | 0.53 | 2.41 | 0.000 | 0.000 | 0.000 | 1145 | tags=45%, list=18%, signal=53% |
| 2 | HALLMARK\_TGF\_BETA\_SIGNALING | Details ... | 37 | 0.49 | 1.73 | 0.003 | 0.020 | 0.045 | 492 | tags=22%, list=8%, signal=23% |
| 3 | HALLMARK\_TNFA\_SIGNALING\_VIA\_NFKB | Details ... | 86 | 0.42 | 1.70 | 0.004 | 0.019 | 0.064 | 701 | tags=26%, list=11%, signal=28% |
| 4 | HALLMARK\_P53\_PATHWAY | Details ... | 101 | 0.33 | 1.44 | 0.004 | 0.108 | 0.393 | 832 | tags=24%, list=13%, signal=27% |
| 5 | HALLMARK\_KRAS\_SIGNALING\_DN | Details ... | 23 | 0.43 | 1.31 | 0.130 | 0.210 | 0.689 | 1530 | tags=48%, list=24%, signal=63% |
| 6 | HALLMARK\_REACTIVE\_OXYGEN\_SPECIES\_PATHWAY | Details ... | 31 | 0.39 | 1.29 | 0.128 | 0.198 | 0.745 | 649 | tags=29%, list=10%, signal=32% |
| 7 | HALLMARK\_MYOGENESIS | Details ... | 74 | 0.31 | 1.24 | 0.093 | 0.227 | 0.828 | 1215 | tags=34%, list=19%, signal=41% |
| 8 | HALLMARK\_HEME\_METABOLISM | Details ... | 92 | 0.29 | 1.21 | 0.103 | 0.237 | 0.874 | 1355 | tags=30%, list=22%, signal=38% |
| 9 | HALLMARK\_ADIPOGENESIS | Details ... | 119 | 0.28 | 1.21 | 0.100 | 0.214 | 0.880 | 1128 | tags=28%, list=18%, signal=33% |
| 10 | HALLMARK\_UV\_RESPONSE\_DN | Details ... | 79 | 0.26 | 1.09 | 0.291 | 0.391 | 0.986 | 1358 | tags=33%, list=22%, signal=41% |
| 11 | HALLMARK\_INTERFERON\_GAMMA\_RESPONSE | Details ... | 64 | 0.27 | 1.06 | 0.361 | 0.412 | 0.995 | 1013 | tags=25%, list=16%, signal=30% |
| 12 | HALLMARK\_BILE\_ACID\_METABOLISM | Details ... | 42 | 0.30 | 1.04 | 0.356 | 0.407 | 0.997 | 1691 | tags=33%, list=27%, signal=45% |
| 13 | HALLMARK\_INTERFERON\_ALPHA\_RESPONSE | Details ... | 38 | 0.28 | 1.02 | 0.398 | 0.412 | 0.999 | 953 | tags=24%, list=15%, signal=28% |
Table: Gene sets enriched in phenotype **na **[plain text format]****
